# Sound envelope extraction in cochlear nucleus neurons: modulation filterbank and cellular mechanism

**DOI:** 10.1186/1471-2202-14-S1-P312

**Published:** 2013-07-08

**Authors:** Bertrand Fontaine, Luis J Steinberg, José Louisa Peña

**Affiliations:** 1 D. P. Purpura Department of Neuroscience, Albert Einstein College of Medicine, Bronx, New York 1046, USA

## 

Efficiently encoding sound features, e.g. temporal fine structure or envelope, is critical for perception tasks such as sound localization and identification. Whereas the auditory nerve (AN) fibers convey all information transduced at the cochlea, the pathway bifurcates in the cochlear nucleus, the first station after AN. The auditory system of the barn owl is a good example of such functional differentiation, where one subdivision of the cochlear nucleus (Nucleus Angularis, NA) is thought to encode sound intensity information used for interaural intensity difference processing whereas the other (Nucleus Magnocellularis) encodes temporal fine-structure information necessary for interaural time difference processing [1].

The fact that NA responses to frozen broadband sounds are very reproducible on a slow time-scale [2] led to think that this center is also involved in the encoding of the slow-time-varying sound envelope. By using coherence and phase-locking analysis of NA responses to amplitude-modulated broadband sounds, we show that NA indeed encodes the envelope better than AN at the cost of losing the fine -structure information. A detailed analysis of the modulation filters estimated using reverse correlation on the envelope signal shows that NA neurons low-pass or band-pass filter the envelope with time constants of an order of magnitude faster than in higher centers (e.g. [3]). Population-wide there is a heterogeneous distribution of the center frequencies of those filters that does not depend on the best frequency of the neurons. This suggests that NA could implement a bank of modulation filters, each with different center frequencies (up to 600 Hz) and bandwidths.

Because the AN encodes sounds through frequency-selective channels that exhibit low-pass modulation sensitivity the question then arises as to how NA cells, receiving a few (around 5 [4]) AN inputs, can implement band-pass modulation filtering. While most existing models of such temporal modulation rely on delayed inhibition (e.g. [5]), there is no evidence of time-locked GABAergic input to NA. We therefore hypothesize this function can be based on cellular mechanisms. Using a model fitting approach [6] we show that a spiking neuron model including an adaptive threshold based on sodium inactivation [7] can predict the spike timing and the response statistics of NA neurons. In particular we hypothesize that the high-pass filtering effect of the dynamic threshold on the membrane potential, in conjunction with the low-pass filtering properties of the cell, serve as a neural substrate for implementing the observed band-pass filtering properties of AN neurons. Population heterogeneity in the model parameters, especially in the time constant of the threshold dynamics, would ensure to have a bank of filters with different frequency responses. This finding indicates that a basic cellular mechanism, in our case spike threshold adaptation, is sufficient to implement neural band-pass filtering.
